# Bilateral Subdural Hygromas Following Rupture of a Temporal Arachnoid Cyst in a 16-Year-Old Boy: A Case Report

**DOI:** 10.7759/cureus.94886

**Published:** 2025-10-18

**Authors:** Marie Bore

**Affiliations:** 1 Department of Radiology, Université Libre de Bruxelles, Bruxelles, BEL

**Keywords:** adolescents, arachnoid cysts, bilateral subdural hygroma, head trauma, intracranial pressure, magnetic-resonance imaging (mri)

## Abstract

Arachnoid cysts (ACs) are benign, usually asymptomatic malformations; however, following minor head trauma, rupture can result in subdural hygromas, with bilateral involvement being uncommon and management often debated. A previously healthy 16-year-old competitive boxer presented with two weeks of headache, nausea, vomiting, and diplopia after a blunt head impact. Examination revealed a right abducens palsy with early papilledema. Non-contrast head CT demonstrated a left temporal AC complicated by bilateral convexity subdural hygromas and an approximately 5-mm rightward midline shift. Given the stable initial examination, close observation was undertaken; however, with progressive signs of intracranial hypertension, a stepwise surgical strategy was implemented, consisting of right subdural drainage and fenestration of the left temporal cyst. Follow-up MRI, including cine phase-contrast sequences, confirmed communication between the cyst and basal cisterns and documented fluctuating hygroma volumes (resolution on the right with contralateral variation) without lasting neurological deficit. The clinical course was favorable, with resolution of headache and improvement of diplopia; papilledema regressed, and no cerebrospinal fluid shunt was required. This case highlights the clinicoradiological variability of post-traumatic hygromas associated with ACs in adolescents and supports a stepwise, individualized approach that prioritizes vigilant surveillance and targeted intervention, with flow-sensitive MRI helping to substantiate shunt avoidance when the clinical trajectory is improving.

## Introduction

Arachnoid cysts (ACs) account for approximately 1% of intracranial lesions, with a predilection for the middle cranial fossa, particularly in children and adolescents [1,2]. Most ACs are asymptomatic and incidentally discovered; however, complications may follow minor head trauma, including rupture with subdural collections (hygroma or hematoma) [3-5]. Bilateral subdural hygromas in the setting of an AC are uncommon and may present with features of intracranial hypertension in adolescents [6,7]. Cross-sectional imaging is essential, CT for mass-effect screening and MRI to characterize the cyst and delineate subdural collections, with flow-sensitive sequences suggesting cyst-cistern communication and helping monitor post-fenestration patency [3-5,8,9]. Because management remains debated, care is best individualized along a graded pathway from observation to targeted surgery, reserving permanent shunting for refractory cases given the risks of over-drainage [3-5]. Natural history data generally favor conservative or limited surgery when the neurological examination is stable and mass effect is modest [1,3-5]. Within this context, we present a case of a 16-year-old boy with a left temporal AC complicated by bilateral post-traumatic hygromas, emphasizing outcomes under stepwise management.

## Case presentation

Patient information and initial presentation

A 16-year-old boy with no relevant past medical history, a competitive boxer, presented to the emergency department on January 31, 2025, with two weeks of progressive headache, nausea, vomiting, new-onset diplopia, and blurred vision following a blunt head impact during boxing practice.

Clinical findings

Neurological examination revealed a right abducens (cranial nerve VI) palsy with intermittent horizontal diplopia. Funduscopic evaluation demonstrated early papilledema. Vital signs were within normal limits; there were no meningeal signs or focal motor or sensory deficits.

Imaging at presentation

**Figure 1:**
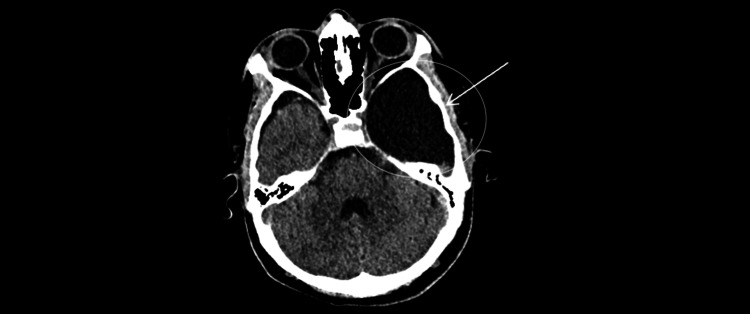
Non-contrast head CT (axial, brain window; W80/L40; 5-mm slices). Large left middle-fossa arachnoid cyst (white arrow) with CSF attenuation, compressing the adjacent temporal lobe; the open circle outlines the region of interest. No acute intracranial hemorrhage is seen at this level.

**Figure 2:**
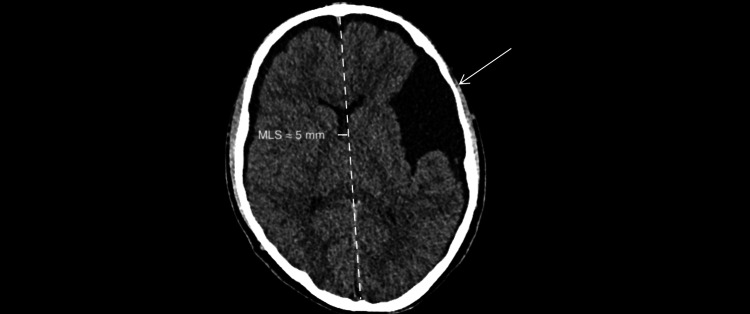
Non-contrast head CT (axial, brain window; W80/L40; 5-mm slices). Approximately 5-mm rightward midline shift, measured perpendicularly from the ideal midline (dashed line) to the displaced septum pellucidum, as indicated by the short perpendicular marker and label. Left middle-fossa arachnoid cyst (arrow).

**Figure 3:**
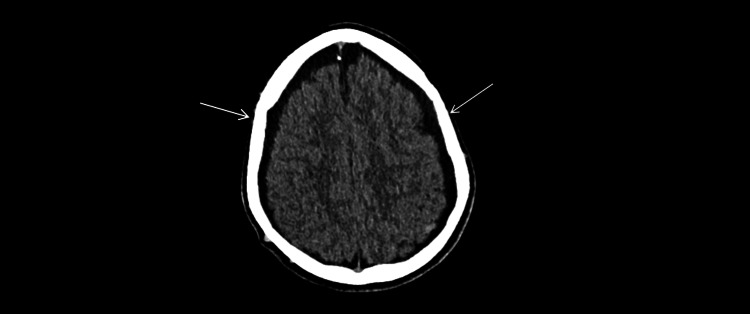
Non-contrast head CT (axial, brain window; W80/L40; 5-mm slices). Thin crescentic subdural fluid collections along the bilateral frontoparietal convexities (arrows), compatible with subdural hygromas.

Non-contrast head CT showed a large left temporal AC in the middle cranial fossa (Figure 1) with bilateral subdural hygromas along the convexities and an approximately 5-mm rightward midline shift. There was no acute hemorrhage (Figures 2-3).

Initial management

Given the stable examination and modest mass effect, the patient was admitted for close observation, head-of-bed elevation, and symptomatic therapy (analgesia and antiemetics as needed). No antithrombotic agents were administered. The neurosurgical team recommended surveillance with short-interval imaging.

Indication for surgery

During February 2025, progressive signs of elevated intracranial pressure (worsening headaches and persistent papilledema) were documented, and a stepwise surgical approach was undertaken: right subdural hygroma drainage followed by fenestration of the left temporal AC (endoscopic/microsurgical according to institutional practice). Imaging triggers for intervention included an approximately 5-mm rightward midline shift with convexity sulcal effacement on presentation CT, followed by persistence and interval enlargement of the left subdural hygroma on short-interval MRI despite observation. In the context of progressive intracranial hypertension, these findings supported a stepwise surgical approach (targeted right subdural drainage and left cyst fenestration).

Early postoperative course and imaging

**Figure 4:**
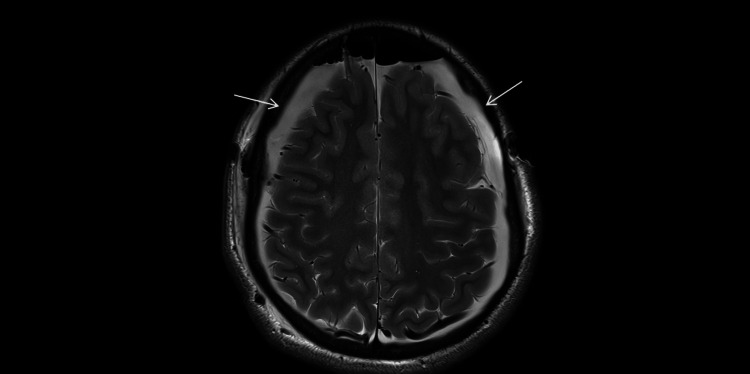
Early postoperative MRI (February 2025; axial T2-weighted; 4-mm slices; 0.5-mm gap). Residual bilateral convexity subdural hygromas (arrows) with slight interval regression compared with the presentation CT (Figure 3).

**Figure 5:**
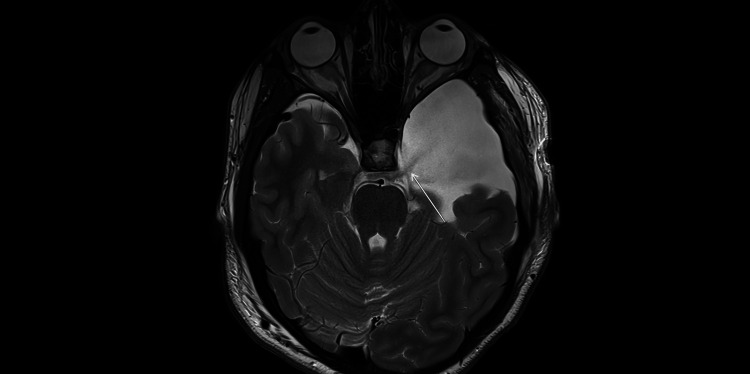
Early postoperative MRI (February 2025; axial T2-weighted; 4-mm slices; 0.5-mm gap). Left middle-fossa arachnoid cyst with a thin CSF-signal channel along its medial wall extending toward the basal cisterns (arrow), compatible with post-fenestration cyst-cistern communication.

An early February 2025 MRI demonstrated partial reduction of the subdural collections with visualized CSF flow between the fenestrated cyst and the basal cisterns, consistent with patent communication (postoperative MRI illustrated in Figures 4-5). Clinically, headaches improved and diplopia became intermittent; papilledema persisted but regressed on neuro-ophthalmologic follow-up.

Interim fluctuation and multidisciplinary decision

**Figure 6:**
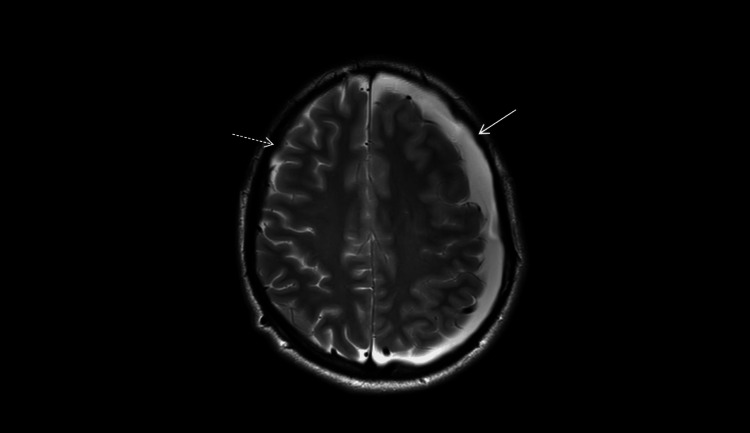
MRI (March 5, 2025; axial T2-weighted; 4-mm slices; 0.5-mm gap). Complete resolution of the right convexity subdural hygroma (dashed arrow) with persistence and interval enlargement on the left (solid arrow), compared with the early postoperative study (Figure 4).

**Figure 7:**
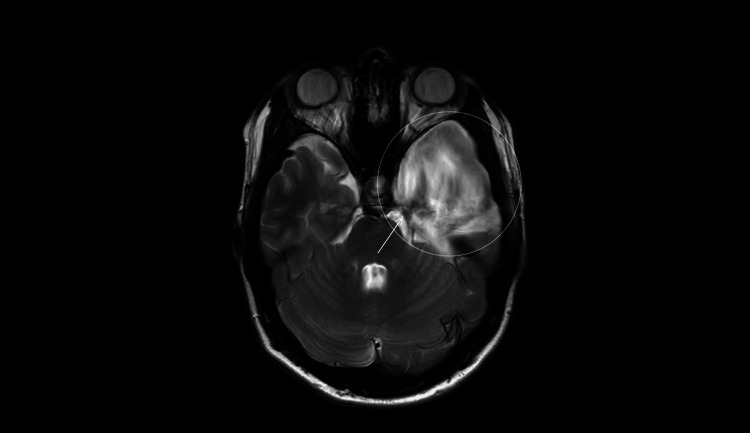
MRI (March 5, 2025; axial T2-weighted; 4-mm slices; 0.5-mm gap). Flow-related signal heterogeneity within the left middle-fossa arachnoid cyst (open circle) along its medial wall (arrow) makes the cyst-basal cistern communication less conspicuous on T2 at this time point.

On March 5, 2025, MRI showed complete resolution of the right hygroma with interval enlargement of the left. On axial T2-weighted images, flow-related signal heterogeneity within the cyst, consistent with turbulent CSF, made the cyst-cistern communication less conspicuous (Figures 6-7). At the March 6 neurosurgical visit, headaches had worsened. Ventriculoperitoneal shunting was considered but deferred in light of the overall clinical trajectory and absence of progressive neurological deficit.

Subsequent evolution

**Figure 8:**
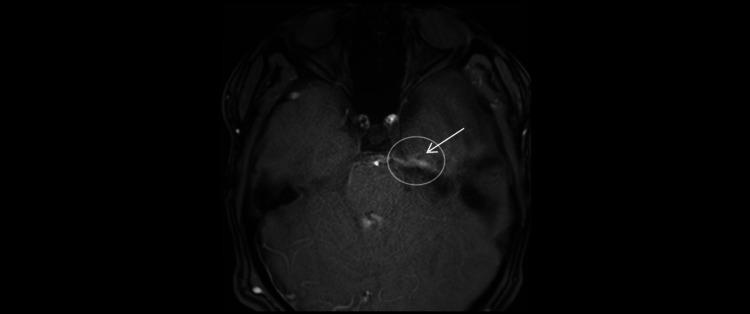
Phase-contrast cine MRI (April 28, 2025; axial 2D PC-MRI; single-slice, 6-8 mm). CSF flow is visualized across the surgical fenestration between the left middle-fossa arachnoid cyst and the basal cisterns (arrow, open circle), confirming patency of the cyst-cistern communication. PC-MRI: Phase Contrast Magnetic Resonance Imaging.

**Figure 9:**
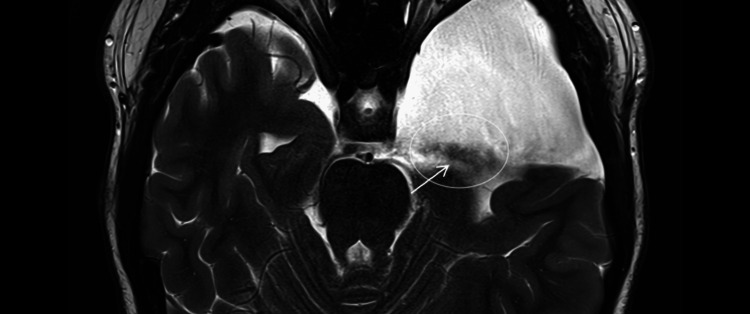
MRI (April 28, 2025; axial T2-weighted; 4-mm slices; 0.5-mm gap). Flow-related artifact along the medial wall of the left middle-fossa arachnoid cyst (arrow, open circle), supporting patency of the surgical fenestration and cyst-basal cistern communication.

By April 3, 2025, headaches had resolved and diplopia improved. The April 28, 2025 MRI confirmed fenestration patency with persistent flow on cine sequences (Figures 8-9). Ophthalmologic and neurosurgical follow-up documented continued improvement; papilledema remained but was regressing.

Status on late follow-up

**Figure 10:**
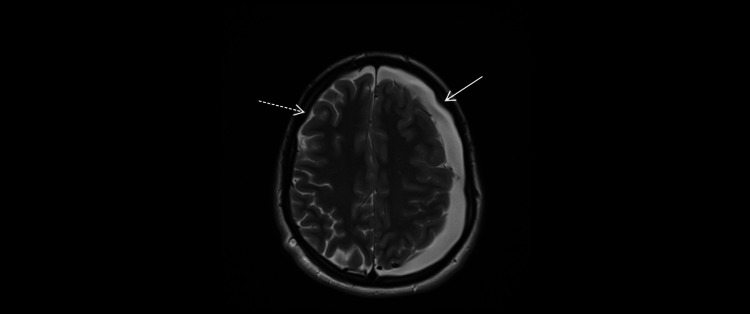
MRI (July 23, 2025; axial T2-weighted; 4-mm slices; 0.5-mm gap). Overall stability of the bilateral convexity subdural hygromas compared with the prior study: the right side remains resolved (dashed arrow), and a residual collection persists on the left without interval increase (solid arrow).

**Figure 11:**
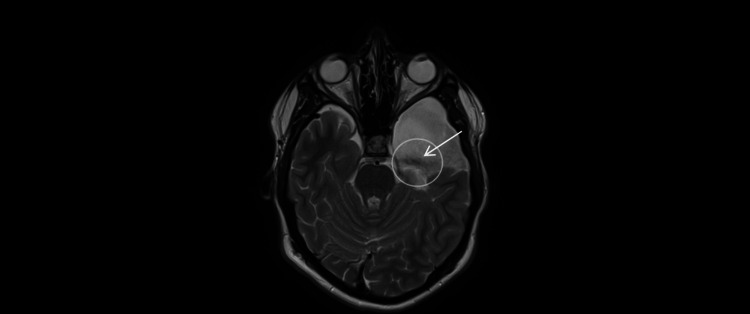
MRI (July 23, 2025; axial T2-weighted; 4-mm slices; 0.5-mm gap). Flow-related artifact along the medial wall of the left middle-fossa arachnoid cyst (arrow, open circle), consistent with persistent patency of the surgical fenestration and cyst-basal cistern communication, in keeping with prior findings.

A July 23, 2025 MRI demonstrated overall stability of the bilateral subdural collections and the left temporal AC compared with the April studies (Figures 10-11). At last follow-up, the patient remained clinically improved, and no cerebrospinal fluid diversion had been performed.

Patient consent

Written informed consent for publication of this case and accompanying images was obtained from the patient’s legal guardian.

## Discussion

ACs of the middle cranial fossa are benign extra-axial lesions commonly seen in children and adolescents. Post-traumatic or spontaneous rupture may allow CSF to enter the subdural space, producing hygromas, sometimes bilateral [10], and a syndrome of intracranial hypertension (ICH) [11]. This sequence, minor head trauma, rupture of a Sylvian AC, subdural hygromas, and ICH, is consistent with findings from pediatric and adolescent series [1,3-5,10]. Several mechanisms likely coexist: tearing of the cyst wall creating communication with the subdural space; slit- or ball-valve dynamics that raise intracystic pressure and promote CSF dissection [12,13]; and, less commonly, a hemorrhagic component accounting for mixed hygroma-hematoma presentations. When a middle-fossa AC ruptures, CSF exits into the subdural compartment. Because the subdural space is a circumferential potential space that communicates across the midline, fluid can redistribute bilaterally despite a unilateral source. Dependent layering and pressure gradients further favor contralateral spread, and transient dysfunction of arachnoid granulations may reduce CSF resorption and help maintain the collections [3-5,10]. These models explain the volumetric fluctuations observed on follow-up, as illustrated in this patient.

Across the literature, management of ACs complicated by subdural hygromas remains controversial, and no evidence-based consensus guidelines are currently available [3-5,10]. In general, surgery is considered for persistent or recurrent hygromas in symptomatic patients, particularly in the setting of ICH, whereas a conservative approach may be reasonable otherwise [14]. This aligns with the benchmark series by Maher CO et al. [3], who proposed a graded strategy: close observation when clinically stable; targeted burr-hole drainage for tense hygromas causing ICH; fenestration of the AC (endoscopic or microsurgical) to re-establish communication with the basal cisterns; and shunting (cysto- or subdural-peritoneal) reserved for refractory or recurrent cases owing to device-related morbidity, including risks of over-drainage and shunt dependence. From an imaging standpoint, surgical consideration is generally warranted for tense or enlarging subdural collections with mass effect (e.g., midline shift or effacement of cortical sulci), for failure of spontaneous regression on serial studies despite observation, or when radiologic progression correlates with refractory clinical ICH [3,14]. These principles are consistent with broader literature advocating individualized management along a graded pathway [4,15,16]. Notably, even some symptomatic cases managed conservatively (watch-and-wait) have demonstrated resolution of symptoms with spontaneous disappearance of subdural collections [3-5,10,15].

In this case, targeted drainage of the right hygroma (symptomatic decompression) combined with fenestration of the left temporal AC fits this restoration-of-flow paradigm [8,9]. Despite initial fluctuations in symptoms and hygroma volumes, shunting was ultimately avoided in light of progressive clinical improvement (including abducens palsy and headache), overall imaging stability, and MRI-demonstrated patency of the fenestration [1,3-5]. This course aligns with reference series and recent reviews showing progressive resolution of subdural collections once CSF flow is restored, reserving shunting for refractory scenarios [3,14].

A key methodological point is the functional assessment of post-fenestration communication [8,9]. Beyond morphology on T2/FLAIR, cine phase-contrast MRI can demonstrate a CSF jet between the cyst and basal cisterns and serves as a non-invasive alternative to cisternography; broader reviews detail technique and clinical utility [8,17]. After rupture, the AC may partially decompress and appear less conspicuous; recognition of secondary signs, e.g., uni- or bilateral convexity subdural hygromas, temporal pole scalloping or widened Sylvian fissure, or a subtle CSF-intensity extra-axial cleft, and confirmatory MRI (T2/FLAIR, DWI) can facilitate diagnosis. In equivocal cases, thin-slice CT and/or cine phase-contrast MRI may help demonstrate cyst-cistern communication [8,9]. In this observation, visualizing flow between the AC and the cisterns (with T2 flow-related heterogeneity and confirmatory cine sequences) was pivotal in sustaining a conservative strategy [3-5,8,9].

Clinical trajectories remain heterogeneous, ranging from spontaneous resolution under surveillance to complications such as chronic subdural hematoma requiring re-intervention; consequently, close clinico-radiological monitoring in the weeks after intervention is warranted, with decisions iteratively guided by clinico-radiological correlation [3-5,10,15].

This report is limited by its single-patient design and the possibility that part of the improvement reflects natural history after minor head trauma. Flow assessment on MRI was largely qualitative (T2 flow-related heterogeneity and cine impressions) without standardized quantification or invasive intracranial pressure measurements. Follow-up duration was moderate, and protocol variations may have affected the conspicuity of the cyst-cistern communication. As a single-patient observation, external generalizability is inherently limited; management should remain individualized. Nonetheless, the combination of headache with abducens palsy and convexity subdural hygromas, especially in the presence of a middle-fossa AC, should prompt consideration of post-rupture hygromas in the differential diagnosis.

Practically, a stepwise, individualized approach appears reasonable: close observation with serial imaging and examinations; targeted subdural drainage for symptomatic decompression; and cyst fenestration to restore CSF pathways, while avoiding premature shunting. Non-invasive MRI markers (cine flow signals, T2 flow-related artifacts) can support conservative management when the clinical trajectory is improving. Multidisciplinary follow-up and temporary avoidance of contact sports are advisable.

## Conclusions

This case demonstrates that bilateral subdural hygromas complicating a temporal AC after minor head trauma in an adolescent can resolve under a stepwise, conservative-to-targeted surgical strategy without cerebrospinal fluid diversion. Management should be guided by clinico-radiological correlation and serial MRI, with demonstration of cyst-basal cistern communication, including cine phase-contrast MRI, as an imaging biomarker of fenestration patency. These findings support individualized, multidisciplinary care with neuro-ophthalmologic follow-up until resolution of intracranial hypertension. While not generalizable, these combined imaging and clinical features may raise suspicion for AC rupture with subdural hygromas in adolescents presenting with headache and abducens palsy, thereby guiding targeted imaging and stepwise management.
